# Care requirements for clients who present after rape and clients who presented after consensual sex as a minor at a clinic in Harare, Zimbabwe, from 2011 to 2014

**DOI:** 10.1371/journal.pone.0184634

**Published:** 2017-09-21

**Authors:** Rebecca E. Harrison, Linda Pearson, Michael Vere, Prosper Chonzi, Brian Tafadzwa Hove, Sharon Mabaya, Margaret Chigwamba, Juliana Nhamburo, Juliet Gura, An Vandeborne, Sandra Simons, Daphne Lagrou, Eva De Plecker, Rafael Van den Bergh

**Affiliations:** 1 MSF-OCB, Harare, Zimbabwe; 2 Mbare Polyclinic, MSF-OCB, Harare, Zimbabwe; 3 City Health, Harare, Zimbabwe; 4 Mbare Polyclinic, MOHCC Harare, Zimbabwe; 5 MSF-OCB, Brussels, Belgium; UNITED STATES

## Abstract

**Study goals:**

To describe the differences between clients presenting after rape and clients who have consented to sex as a minor to an SGBV clinic in Harare, Zimbabwe, and how these differences affect their care requirements.

**Background:**

Adolescents and adults presenting at the specialized Sexual and Gender Based Violence clinic in Harare are offered a standardised package of free medical and psychosocial care. Zimbabwe has an HIV prevalence of 14%, so prevention of HIV infection using PEP for those that present within 72 hours is a key part of the response. STI treatment, emergency contraceptive pills, referral for termination of pregnancy, psychological, social and legal support is also provided.

**Methods:**

This is a retrospective descriptive study of routine programmatic data collected at the Edith Opperman polyclinic in Mbare SGBV clinic from 2011 to 2014. Chi-square tests and logistic regression were used to describe the different experiences and the differences in uptake of care between clients presenting for rape compared to those who consented to sex as a minor.

**Results:**

During the study period a total of 3617 clients presented to the clinic. 2242 (62%) sought care after rape, 602 (17%) for having consented to sex as a minor and 395 (11%) for suspected sexual abuse. 1615 (45%) of people presenting were 12–15 year olds. Minors who consented to sex compared to survivors of rape were less likely to report within 72 hours– 156 (26%) vs 894 (40%) p<0.001; less likely to report that they delayed due to fear– 68 (17%) vs 472 (40%) p<0.001, less likely to have experienced accompanying violence– 9 (1%) vs 176 (8%) p<0.001 or physical trauma—34 (6%) vs 427 (19%) p<0.001; and less likely to display psychological symptoms at presentation 51 (8%) vs 411 (18%) p<0.001. Minors who consented to sex compared to those who were raped were less likely to start PEP if eligible—123 (80%) vs 751 (93%) p<0.001, less likely to take emergency contraceptives if eligible—125 (81%) vs 598 (88%) p<0.001, more likely to be pregnant– 132 (23%) vs 241 (15%) p<0.001; less likely to request a termination of pregnancy if pregnant—10 (8%) vs 74 (31%) p<0.001; and less likely to come for at least one follow up 281 (47%) vs 1304 (58%) p<0.001.

**Conclusion:**

The experiences of those who consent to sex as a minor and those that have experienced forced sex were very different. The standardised SGBV medical response does not fully meet the needs to protect minors who have consented to sex from HIV or unwanted pregnancies. Clients who present for having consented to sex as a minor might benefit more from being offered long-term family planning or being assessed as a sero-discordant couple rather than simply PEP and ECP as is relevant for clients who have been raped. More provision of health care is needed for minors to ensure they have access to enough information and protection from HIV, other STIs and unwanted pregnancy, before they decide to engage in sexual intercourse, rather than as an emergency at an SGBV clinic.

## Introduction

Adolescents, and especially young girls, are disproportionately affected by sexual and reproductive health problems [1–3]. In sub-Saharan Africa, young females were estimated by UNAIDS to contribute one in four new HIV infections, and young women were estimated to be infected with HIV 5–7 years earlier than men [4]. Age of consent laws are in place in many countries to protect minors from the dangers of engaging in sexual intercourse at a young age, and punish those that have sex with minors. Individual countries have different age limits for consent to sex, varying from 12 to 18 across much of the world [5]. Age of consent laws are based on the notion that adolescents are less capable of making rational decisions than adults, though there is a lack of consensus and clear evidence on this matter and on the age threshold at which adolescents are competent to make full decisions [6].

However, while such laws may protect individuals who are indeed not yet competent to make decisions on sexual behaviour, they can have an adverse effect on the sexual and reproductive health of the adolescents too[7]. By criminalising sex for adolescents, access to contraceptive care, including dual / triple protection (both before and after sexual intercourse), and termination of pregnancy services is compromised [8], with potentially severe consequences such as unsafe abortions or complications during childbirth [9]. As a result of strictly enforced age of consent laws in some settings, and as shown by this analysis, adolescents engaging in consensual sexual intercourse may end up presenting to sexual violence programmes, as parents or legal guardians may prefer to report a sexual act as rape. However, as such programmes are geared towards the care for victims of forced sex, they may not be ideally suited for the needs of minors engaging in consensual sex.

Zimbabwe is such a country with legislation on age of consent and sexual intercourse with adolescents. Zimbabwean law specifies that a child under 12 is totally incapable of consenting to sex, and anyone found to have had sex with a child of this age will be charged with rape and can be sentenced to life imprisonment [10]. For minors between 12 and 15, charges depend on the consent of the minor, whether the minor was married, and/or whether the sexual act would be considered indecent by a reasonable person. Further confusion is added by the process of aligning the laws to the new constitution [11], which further codifies the ages at which individuals are allowed to marry. As a consequence of Zimbabwean law, sexual violence clinics in the country—such as the Edith Opperman clinic in Mbare, operated by Médecins Sans Frontières (MSF)–are confronted with high numbers of adolescents presenting after having consented to sex rather than after forced sex. While this study does not aim to trivialize the potentially life-long consequences of starting sexual intercourse too early, we have avoided the umbrella term of ‘statutory rape’, in order to distinguish individuals who reported to have had consensual sex as a minor, and those that experienced forced sex.

This study was conducted to better understand the different characteristics of clients who present for forced sex and for those that consented to sex as a minor, in order to understand the extent to which the care provided by sexual violence clinics to adolescents engaging in consensual sex is suited to their needs. We conducted an analysis of the routinely collected data of clients presenting at the Edith Opperman sexual violence clinic, in order to compare 1) the client characteristics, 2) the incident characteristics, 3) factors affecting timely presentation and 4) the medical & legal management of clients presenting for rape and clients who have consented to sex as a minor.

## Materials and methods

### Ethics statement

This study met the Médecins Sans Frontières’ Ethics Review Board-approved criteria for analysis of routinely collected program data. Approval was also granted by Harare City Health Institutional Review Board.

### Study design

This was a retrospective descriptive study of routine programmatic data collected at the Edith Opperman polyclinic, in Mbare Sexual and Gender-Based Violence (SGBV) clinic. All people who sought care between September 2011 and December 2014 were included.

### Study setting—General

The median age at first sex in Zimbabwe was reported at the last Demographic and Health Survey 2010–2011 (DHS) to be 18.9 for women and 20.6 for men, though for women this age varied by level of education [12]. The median age at first sex for women aged 25–49 with no education was 16.9 compared to 22.3 for those with more than secondary education. This would indicate that close to 50% of people with no education had sex for the first time under the legal age of 16. For men, the difference between the most educated and the least educated was quite minimal. 8.6% of the country’s population had no education, compared to 2.8% that had more than secondary education. According to the latest DHS 99% of al Zimbabweans have heard of at least one modern contraceptive method however only 12% of 15–19 years report using a modern family planning method, compared to 67% in the 30–35 age group [12]. For the most part (73%) 15–49 year old women get family planning methods from government health centres who are legally allowed to provide any method to minors [12].

According to Zimbabwean law, sexual acts with a child under 12 are always considered rape and can be sentenced to life imprisonment [10]. For minors aged 12 to 15, the capacity to consent is recognised: in the case of consent, the charge can be “sexual intercourse or performing indecent acts with a young person” if the act is extra-marital or if it would be considered indecent by a reasonable person, whether in or outside of marriage. There has been considerable discussion in Zimbabwe regarding increasing the age limit for recognising the capacity to consent, though it has yet to be passed in law. For the purposes of this study the definition of an adolescent or a minor is 12–15 years old, as this is the age bracket where someone having sex with a minor can be charged with an indecent act with a young person, rather than rape if consent can be proved. The term child in this document is used to refer to people from the age of 0–18, in line with its use in the Zimbabwean constitution. The definitions of the events as used in this study (as opposed to the legal terms relating to specific crimes in Zimbabwe) are outlined in Box 1

**Box 1**

**Rape**—refers to cases of penetrative sexual intercourse of a women who did not consent

**Sexual intercourse with a minor**—Sex with a minor refers to consensual sex between a minor between 12 and 15 years with another minor or with an adult. The term statutory rape has not been used in this document, in order to make a clear distinction between forced sex, and sex with a consenting minor.

**Suspect cases**—Suspect case refers to cases where the minor denies any assault (cases where minors who are suspected of having a sexual relationship with a ‘boyfriend’ by a parent or guardian and are bought to the clinic for examination), or if a client is unable to give a history (in the case of very young children, or because of physical or mental incapacity) and no evidence of sexual activity is found by the nurse.

**Aggravated indecent assault**—where a female has sex with a male against his will—these cases usually involve groups of women and weapons, or older women with younger boys. Men cannot be raped according to the national law, though women can be subjected to aggravated indecent assault. But for the purposes of classification of this study, only men are given this classification. Aggravated indecent assault in the law refers to unlawful and intentional assault that involves penetration of a part of either the victim’s or the perpetrator’s body.

**Sodomy**—refers to non-consensual anal sexual intercourse, and always refers to a male victim and male perpetrator.

**Sexual touching or attempted rape**—where a person experiences attempted rape, or other sexual advances that do not include penetration

**Non-sexual aggression**—refers to violence that is not sexual in nature. There are very few of these cases as the clinic did not specialise in this type of case

### Study setting—Specific

The Sexual and Gender Based Violence clinic based at Edith Opperman Polyclinic is situated in Mbare, Harare. Mbare was the first suburb of Harare, the capital city of Zimbabwe and was built in 1907 and is now a high-density suburb. The overall population of Harare city at the last census was estimated to be around 1.6 million and estimations of the population living in Mbare vary between 142,195 and 84,168. The HIV prevalence rate in Zimbabwe is currently estimated to be around 14%, and higher in urban areas such as Mbare.

Mbare is characterized by a young mobile population, the bustle of the markets, the transport hub, a vibrant music scene, and is home to the second biggest football stadium after the national sport stadium. While no formal data is available on unemployment rates in this part of the city it is likely that the majority of the population do not have formal employment (as is the case nationwide) and are dependent on informal trading for an income. Living conditions are generally poor with overcrowding, frequent cuts in the supply of water and electricity, poor roads, lighting and lack of basic infrastructure. Access to education and healthcare is not free, and is unaffordable for some. According to the 2010–2011 DHS, 90% of females in the highest wealth quintile have completed secondary schooling and only 41% from the lowest quintile completed secondary school. In 2010–11, 26% of minors aged 13–18 of the lowest wealth quintile were currently estimated to be in school compared to 69% of those in the highest, indicating that the situation is getting worse for poorer minors.

### Sexual violence programme in Mbare Polyclinic

MSF opened a “vertical” sexual violence clinic, run by nurses, in Mbare, in conjunction with City Health in September 2011, with the objective of providing comprehensive care to survivors of SGBV. To make prosecution of sexual abuse cases more effective, the Zimbabwean law was updated and, since 2006, allows and encourages trained nurses as well as doctors to complete medical affidavits. People come to the clinic from all over Harare, and sometimes from other parts of the country. Care is sought for the following problems—rape, having consented to sex as a minor, aggravated indecent assault, attempted rape, non-sexual aggression, sodomy and suspected sexual activity (see Box 1). The majority of clients seeking care at the clinic are women, and the majority are under 16. Perpetrators tend to be male, and are usually over 18.

The SGBV clinic follows the Ministry of Health and Child Care (MOHCC) national protocol on care for survivors of sexual violence. The people who attend the clinic are offered free medical care, counselling, and social support, and are referred for psychological, social and legal support as relevant. In brief, clients on presentation are received by a nurse counsellor and a thorough medical history is taken, and a first counselling session and rapid HIV, syphilis and pregnancy testing are done as required. The initial response differs depending on how much time has passed since the assault (within 72 hrs, from 72–120 hrs and above 120 hrs). The key differences are that Post Exposure Prophylaxis (PEP) is offered to those who have presented within 72 hours of the last sexual act, and emergency contraceptives are provided to those who present within 120 hours.

The clinic works closely with the police to ensure prompt referral of clients, particularly the Victim Friendly Unit, The clinic also works in close collaboration with the Victim Friendly Courts, the Department of Child Welfare and Protection Services (DCWPS), City Health Harare and MOHCC to provide the necessary referrals to government departments or to other NGOs according to the protocol on multi-sectoral management of sexual abuse in Zimbabwe. Health promotion activities have also been conducted as part of MSF’s project to raise awareness of SGBV in the community, and the importance of seeking immediate medical care.

### Data collection

A standardised MSF Excel-based electronic database designed for SGBV and adapted to the Zimbabwe context with case definitions was used. Client’s data was entered into the database directly from the client file by a nurse from the project. A client file is filled out by the consulting nurse during the initial and each follow up visit. This contains a written description of the event itself, a medical history, details of the medical forensic examination, and information on all treatment given and referrals provided to the client. All client files were given a sequentially produced ID number, which was also entered into the database. The unique ID meant that the client could not be identified from the database without reference to the client file. There were no other features in the file which would allow identification of the client: for example, place of abode was described using ward or district. The files were kept in a locked storage room. Specific training was given on how to transfer the data from the client file into the database. Monitoring and verification of the file and the electronic database was carried out on a regular basis to ensure correct data collection. Further data cleaning for internal consistency was carried out for this study, and verification with the client file was conducted where necessary.

### Data analysis

All analysis was carried out in STATA version 11 (StataCorp, College Station, TX, USA). Summary descriptive statistics were calculated, and differences between adults and minors who consented to sex as a minor compared to those that were raped have been highlighted. Chi-square tests were carried out and their respective p values are reported. Fisher’s exact tests were utilised where appropriate and are indicated with *** in the tables.

Logistic regression was carried out to better understand the factors that affect presentation within 72 hours. Model building was performed using forward stepwise regression modelling. The procedure for the stepwise model building was to start with a model with the most significant explanatory variable, and to add the next most significant variable in a stepwise manner. If the most significant term was “significant”, it was added to the model and the next most significant variable was tested. Repeatedly, if the most-significant excluded term was “significant”, it was added and re-estimation done; if the least-significant included term was “non-significant”, it was removed and re-estimation done, until neither was possible. Tests for interaction were carried out for the variable “consent”, but none found to be significant. No client could be identified from the dataset during the analysis.

## Results

### Client characteristics

**Fig 1:**
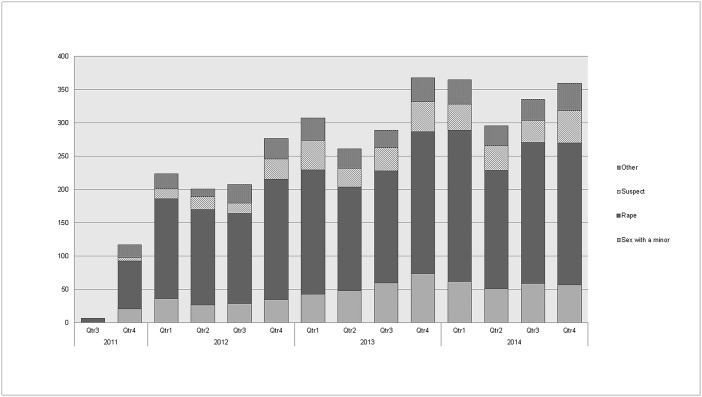
Number new SGBV clients per quarter by reason for visit, Mbare SGBV clinic (09/2011–12/2014).

The number of clients seen at the Mbare SGBV clinic has steadily increased since its opening in September 2011 (Fig 1), particularly amongst 12–15 year olds. Over the entire study period, 3617 individuals were seen at the clinic, 242 (7%) were male and 3375 (93%) were female. 1615 were aged 12–15 (45%), 1071 (30%) aged 16 and older and 931 (26%) below 12. Of the 1615 clients aged 12–15, 707 (44%) reported for rape and 599 (37%) presented as clients who consented to sex as minor, where the girl (only very few cases were boys) involved did not see herself as having been raped and often saw the man involved as her boyfriend. 217 (13%) of 12–15 year olds were brought for consultation by a guardian as “suspect case” for sexual activity, often against their will. This classification of “suspect case” is given if a nurse finds no evidence of sexual activity, after discussion and examination (cf. Box 1). Rape was the most common reason that clients presented to the clinic: 2242 (62%) of all clients and 986 (92%) of clients in the over 16 age group.

#### Incident characteristics

Table 1 describes the profiles and ages of the perpetrator(s). Clients who consented to sex as a minor, were most likely to describe the person as their boyfriend– 550 (91%), whilst those that were raped were most likely to be raped by a known civilian (879; 39%), a family member (468; 21%), or boyfriend (444; 20%). The ages of the “perpetrators”/sexual partners of the consenting minors were largely adults in the 19–30 age group—391 (65%). Only 96 (16%) of the cases reported were with partners of similar age (15–18).

**Table 1:** Incident characteristics, Mbare SGBV clinic (09/2011–12/2014).

	All clients		Consented to sex as a minor		Rape	
	N	%	N	%	N	%
Total	3617		602	17%	2242	62%
Profile of perpetrator			***p<0.001			
Known civilian	1208	33%	33	5%	923	41%
Unknown civilian	460	13%	7	1%	391	17%
Family member	546	15%	4	1%	468	21%
Boyfriend	1030	28%	550	91%	444	20%
Not known	373	10%	8	1%	16	1%
Age of perpetrator			P<0.001			
<14	274	8%	5	1%	164	7%
15–18	373	10%	96	16%	224	10%
19–30	1269	35%	391	65%	778	35%
>30	371	13%	15	3%	388	17%
Unknown	1246	34%	95	16%	688	31%
Weapon use			***p<0.001			
Armed assault	115	3%	1	<1%	105	5%
Associated violence*			p<0.001			
Associated violence	201	6%	9	1%	176	8%
Physical trauma**			p<0.001			
Physical trauma	553	15%	34	6%	427	19%
Hymen tear	2094	58%	548	91%	1168	72%
Psychological symptoms			p<0.001			
Any psychological symptom	517	14%	51	8%	411	18%

* Including beatings, robbery, sexual exploitation but not whether or not attacked was armed

**includes lacerations, bruises, burns, anal tears and other injuries but not including

*** Fisher’s exact test

A low incidence of other violence associated with sexual assault was observed (Table 1). When comparing the clients who consented to sex as a minor and those who were raped, 1 (<1%) vs. 105 (5%) underwent an armed assault, 9 (1%) vs. 176 (8%) reported that they experienced other violence and 34 (6%) vs. 427 (19%) presented physical trauma to their bodies, other than hymeneal tears. Similarly, 51 (8%) of those that consented to sex as a minor displayed psychological symptoms compared to 411 (18%) of those who were raped.

### Presentation time and follow up

The majority of survivors are referred to the clinic by the police 3386 (94%). Timely presentation (within 72 hours of the last event) was significantly higher for rape cases compared to those who had sex as a minor (40% vs. 26%—Table 2). Clients who arrived after 72 hours of the last event were asked why they were not able to come sooner: those who gave reasons are shown in Table 2; 1773 clients gave reasons and the most common reason was fear 602 (34%). The ‘caretaker found out later’ was reported by 519 (20%) to be the reason for delay. The reason ‘other’ was given by 519 (29%) clients, rising to 351 (63%) amongst those who consented to sex as a minor. ‘Other’ was commonly registered for minors who see themselves as in a consenting relationship and saw no reason to come to the clinic earlier. Family negotiation (150; 8%) was often linked to cases where the perpetrator was thought of as having taken the girl’s virginity and negotiations were usually aimed at marriage or payment of compensation. Institutional factors, i.e. lack of knowledge about the availability of medical treatment for rape (79; 4%), delay by the police (53; 3%), lack of access to health structure (21; 1%), were also recorded to a lesser extent. Overall, only 1796 (50%) of the clients came back for at least one follow-up visit (see Table 2). Those who were raped had higher follow-up rates than those who consented to sex as a minor—1304 (58%) vs. 281 (47%) came for at least one follow-up.

**Table 2:** Time to presentation and follow-up, Mbare SGBV clinic (09/11–12/2014).

	All clients		Consented to sex as a minor		Rape	
	N	%	N	%	N	%
Time passed before presenting for treatment			p<0.001			
< 72 h	1290	36%	156	26%	894	40%
72–120 h	259	7%	52	9%	159	7%
6 days—1 month	601	17%	125	21%	389	17%
1 month—1 year	904	25%	237	39%	578	26%
>1 year—5 years	166	5%	16	3%	127	6%
Not entered	397	11%	16	3%	95	4%
Number reporting >72 hours	2327	64%	446	74%	1348	60%
Total reasons given for delay (% of those >72 hrs)	1773	76%	396	89%	1166	86%
Cause of delay in consultation			p<0.001			
Lack of knowledge of treatments	79	4%	9	2%	58	5%
Fear	602	34%	68	17%	472	40%
Caretaker found out later	348	20%	42	11%	229	20%
Delayed by the police	53	3%	6	2%	42	4%
Family negotiation	150	8%	18	5%	125	11%
Other	520	29%	253	63%	240	19%
Follow-up	3617		p<0.001			
No follow-up visits	1796	50%	321	53%	938	42%
At least one return visit	1821	50%	281	47%	1304	58%
At least second return visit	1050	29%	145	24%	788	35%
At least third return visit	562	16%	79	13%	441	20%
At least fourth return visit	214	6%	25	4%	175	8%

### Factors affecting presentation within 72 hours

Associations between different client/incident characteristics and presentation within 72 hours were assessed through logistic regression (Table 3). The most significant independent factor was being sexually abused by an unknown person compared to having intercourse (either consensual or not) with a boyfriend (adjusted OR for timely presentation: 3.89, p<0.001). The second most significant factor was having been referred by the police (adjusted OR 3.07, p<0.001). Presenting with an injury (adjusted OR 2.90, p<0.001) having experienced violence (adjusted OR 1.76, p = 0.004), and being over 16 (adjusted OR 1.35, p = 0.006) were also independently associated with presenting within 72 hours. On the other hand, having STI symptoms (adjusted OR 0.25, p<0.001) and being pregnant (adjusted OR 0.19, p<0.001) were associated with presenting after 72 hours. Whether or not consent was reported, was not a significant predictor of reporting within 72 hours after adjustment for age, possibly as consequence of collinearity.

**Table 3:** Factors associated with presentation within 72 hours after an incident of sexual violence, Mbare SGBV clinic (09/11–12/2014).

Characteristic	Odds Ratio	P value	Adjusted Odds Ratio	P value
**Age group**				
12–15	1.00		1.00	
16+	1.94	<0.001	1.35	**0.006**
**Sex**				
Male	1.00			
Female	1.14	0.353	-	
**Associated Violence**				
None	1.00		1.00	
Violence	3.56	<0.001	1.76	**0.004**
**Armed Perpetrator**				
Not armed	1.00			
Armed	2.70	<0.001	-	**-**
**STI symptoms**				
None	1.00		1.00	
Symptoms of STI	0.45	<0.001	0.35	**<0.001**
**Physical injury**				
None	1.00		1.00	
Physical injury recorded	2.91	<0.001	2.90	**<0.001**
**Psychological symptoms**				
None	1.00			
Psychological symptoms	1.46	<0.001	-	-
**Consent**				
No consent reported	1.00		1.00	
Consent reported	0.50	<0.001	0.83	0.208
**Pregnancy**				
Not pregnant	1.00		1.00	
Pregnant	0.21	<0.001	0.19	**<0.001**
**Year**				
2011–2013	1.00			
2014	1.16	0.037	-	**-**
**Referred by**				
Anything other than police	1.00		1.00	
Police	1.85	0.190	3.07	**<0.001**
**Type of Event**				
Rape	1.90	<0.001	-	-
Consensual sex as a minor	1.00		-	-
**Number of aggressors**				
1	1.00		-	-
2+	1.64	0.003	-	-
Unknown	0.39	<0.001	-	-
**Type of aggressor**				
Boyfriend	1.00		1.00	
Family member	0.87	0.252	0.61	**0.007**
Known civilian	1.71	<0.001	1.11	0.410
Unknown civilian	5.22	<0.001	3.89	**<0.001**
Not known	0.57	<0.001	1.93	0.428

### Medical and legal management

Treatment characteristics are indicated in Table 4. 3456 (96%) of all clients presenting to the clinic were tested for HIV at the first visit and 21 (1%) refused to be tested. 90 (4%) of those who were raped knew they were HIV positive at first contact compared to 9 (1%) of those who had consented to sex as a minor. Of those that had an HIV test, a further 121 (3%) tested positive at the first visit, which again was more common amongst those who were raped (98; 4%) compared to those who had consented to sex as a minor (11; 2%). 971 (82%) of the clients eligible for PEP (HIV-negative, presenting within 72 hours) were recorded as having been given PEP. This rose to 751 (93%) amongst clients who were raped, and dropped to 123 (80%) of those who had consented to sex as a minor. Clients are given the full dose of PEP on the first visit to avoid treatment interruption. To note is that the national protocol does not allow for variations according to the nature of the crime or of the ongoing nature of the sexual relationship—e.g. PEP and ECP is provided to all clients reporting within 72 hours. Only 136 (14%) of those who started PEP had a recorded follow-up visit after 3 months (90 days). There was no difference between 90 day follow-up rates between those who were raped or those who had consented to sex as a minor. Of those that returned, 115 (85%) had a documented HIV test, and only one of these was positive.

**Table 4:** Medical-legal management Mbare SGBV clinic (09/11–12/2014).

	All clients		Consented to sex as a minor		Rape		
	N	%	N	%	N	%	P value
HIV testing at first contact							
Turned down	21	1%	2	0%	11	0%	***p<0.0001
Test not available	10	0%	3	0%	3	0%	
Status already known	112	3%	9	1%	90	4%	
Postponed to a later date	10	0%	0	0%	6	0%	
Consented to testing	3456	96%	586	97%	2128	95%	
Not documented	8	0%	2	0%	4	0%	
PEP Prophylaxis							
Eligible (all clients presenting within 72h who are not HIV+)	1189	33%	153	25%	810	36%	
Started (out of eligible)	971	82%	123	80%	751	93%	p<0.001
Completed (out of started)	900	93%	119	97%	702	93%	p = 0.159
HIV testing at follow up							
Follow up recorded >3 months (out of those given PEP)	136	14%	15	12%	101	13%	p = 0.450
HIV test carried out > 3 months (out of those FU)	115	85%	14	93%	84	83%	***p = 0.760
HIV test positive > 3 months (out of tested)	1	1%	0	0%	1	1%	
HIV test negative > 3 months (out of tested)	114	99%	14	100%	83	99%	
HIV testing at any visit							
Positive	239	7%	21	3%	193	9%	***p<0.001
HIV status already known	112	3%	9	1%	90	4%	
Positive at first visit	121	3%	11	2%	98	4%	
Positive at follow up	6	0%	1	0%	5	0%	
Negative	3333	92%	574	95%	2028	90%	
Emergency Contraception							
Eligible (all female who experienced rape age 12–45 presenting within 5 days)	906	25%	155	26%	677	30%	
Started (out of eligible)	728	80%	125	81%	598	88%	p<0.001
Pregnancy test							
Eligible (all female clients age 12–45)	2565	71%	598	100%	1680	75%	
Test done (out of eligible)	2426	95%	581	97%	1585	94%	p = 0.073
Test positive (out of done)	376	15%	132	23%	241	15%	p<0.001
TOP requested (out of pregnant)	86	23%	10	8%	74	31%	p<0.001
Referral to ANC	228	6%	95	16%	130	6%	<0.001
Referral to pregnancy support	43	1%	13	2%	29	1%	P = 0.118
Out of Court Settlement							
Case recorded settled out of court at last FU (out of those with at least 1 FU)	137	8%	31	11%	92	7%	p<0.001
Case not settled out of court at last FU (out of those with at least 1 FU)	1363	75%	181	64%	1010	77%	
Unknown if case settled out of court (out of those with at least 1 FU)	321	18%	69	25%	202	15%	
Court Settlement							
Case in court at last follow up (out of those with at least 1 FU)	712	39%	103	37%	534	41%	p = 0.368
Case not in court (out of those with at least 1 FU)	929	51%	148	53%	651	50%	
Unknown court outcome (out of those with at least 1 FU)	180	10%	30	11%	119	9%	
Referral for other services							
MSF Social worker	432	12%	77	13%	285	13%	p = 0.959
Antenatal Care	228	6%	95	16%	130	6%	p<0.001
External referral HIV	107	3%	9	1%	87	4%	p = 0.004

*** Fisher’s exact test

In total 376 (15%) women were found to be pregnant either as a result of the rape or due to a pre-existing pregnancy, and 86 (23%) of them opted for termination of pregnancy (TOP—Table 4). Among those eligible for emergency contraception we noted that clients who consented to sex as a minor were less likely to take emergency contraception (125; 81%) than those who were raped (598; 88%), and were more likely to be pregnant (132; 23%) and less likely to request a termination of pregnancy (10; 8%) than those who were raped (respectively 241; 15% and 74; 31%). Data on conducting TOP was incomplete, as this was done by referral and follow-up of cases was not always possible.

In terms of legal management, at the last follow-up visit overall 137 (8%) clients reported an out of court settlement and 712 (39%) reported that the case was in court. Those who consented to sex as a minor were more likely 31 (11%) than those who were raped 92 (7%) to have an out of court settlement, and were slightly less likely (103; 37%) to still have their case in court at the last follow-up visit than those who were raped (535; 41%), though the difference was not statistically significant. Many cases were lost to follow up before any kind of settlement of the case was reported, and this information is consequently not complete.

12% of all clients, and 13% of both rape victims and minors who consented to sex, were referred to the MSF social worker service, which collaborated with the DCWPS. From there, clients were referred onwards for a variety of other services such as legal advice, protection in a safe house, pregnancy support, etc. 95 (16%) clients who had consented to sex as a minor, compared to 130 (6%) of those who were raped, were referred for antenatal care, and 9 (1%) compared to 87 (4%) were referred for external HIV services respectively.

## Discussion

Whilst the majority of clients coming to the clinic, and receiving the same standardised package of care, consulted for rape (62%), a substantial number also reported for having consented to sex as a minor (17%), or for suspected sexual activity (11%). The aim of the Zimbabwean law pertaining to consent to sex, and the standard medical package of care provided through SGBV services, is to offer protection to those who experience incidents such as rape or sex under the legal age of consent. However, this study has identified a number of key differences in the programmatic management of individuals who underwent rape and minors who consented to sex, with the latter group being less likely to have experienced accompanying violence or physical trauma (other than hymen tears), and being less likely to display psychological symptoms at presentation. Whilst the psychological evidence is not conclusive about adolescents’ capacity to make decisions to avoid risk, others have argued that the belief that adolescents are not mature enough to consent to sex is a barrier for adolescents to access contraceptive care, and termination of pregnancy services that could benefit them [2]. Our results support the latter notion, and suggest that the gaps in care provision for the differing events might require different solutions.

A principle risk for both groups in a setting like Zimbabwe, with a national prevalence of HIV of 14%, is acquiring HIV. PEP is the standard measure to prevent this, and is provided regardless of the nature of the event and regardless of the complex situations that many of these incidents occurred within. For PEP to be effective, the person needs to report within 72 hours of the event. However, only 40% of those who were raped and 26% of those who consented to sex as a minor presented to the clinic within 72 hours. The regression analysis shows that presentation within 72 hours is considerably better for those that report some sort of physical violence or injury, and/or for those who were assaulted by an unknown civilian or were referred through the police. However a minority of perpetrators were unknown to the client (1% of minors who consented to sex, and 17% of clients who were raped). For those who were raped, the most commonly reported reason for delaying seeking help was ‘fear’. This fear could be the result of a direct threat from the perpetrator, even in cases when this was a family member, a boyfriend, or someone well-known to the victim. It could also be a more indirect fear, related to disclosure of the SGBV event, Other potential underlying factors could be the fear that help will not be confidential and that police, health providers or people around them might not believe them, or that their family and friends might blame them for ‘asking for it’. The risk of the perpetrator being found not guilty in court is likely to increase this fear. For other cases, when the perpetrator is the client’s father, due to the respect given to the father the client might not disclose out of fear that nobody will believe her. In some cases if the perpetrator was either a provider or the sole provider to the family, the consequences of seeking help might be that the perpetrator would go to jail and would be unable to continue to provide for the family.

The main reason reported for delaying presenting to the clinic for those who consented to sex as a minor was reported to be ‘other’ (63%), which was often related to the fact that the adolescent saw the ‘perpetrator’ as her boyfriend. Whilst this was not recorded in the database, many of the clients presenting to the clinic had sexual intercourse on a number of occasions before presenting for care. For some, the girl involved did not see herself as having been raped and sought treatment only when someone else, usually a relative, discovered the encounter. For others, presentation for treatment is usually preceded by a crisis of some description i.e. pregnancy, the boyfriend becoming violent, learning of a pre-existing marriage, or simply the break-up of the relationship. This reflects a key gap of the standard package of care for minors who consent to sex: for such individuals, care should ideally be sought *before* the event, rather than within 72 hours after an ‘emergency’, so that dual methods of protection can be used—i.e. barrier methods or family planning methods during and before the event and ECP and PEP if necessary afterwards. Encouraging the male to participate in the process for clients who have had consensual sex as a minor could be warranted; to ascertain their HIV and STI status at the earliest stage possible and identify couples that are sero-discordant. Similarly, the incidence of pregnancy at a young age and also STIs indicates that there are gaps in the provision of sexual education for young people, before they consent to sex, enabling them to give full, informed, voluntary consent to an act that they already understand. Many of the female adolescents presenting to the clinic would have benefited from more education on what sex is, and their right to refuse sex, even with their boyfriends. Their male counterparts would benefit from education that it is not inherently their right to have sex with the woman they consider their girlfriend.

The family planning guidelines of Zimbabwe specify that adolescents who are sexually active, even below the age of consent, should be allowed access to the contraceptive method of their choice. This study indicates that more efforts should be made to ensure that the options are discussed with adolescents presenting to clinics, given that a high proportion of minors who have presented to the SGBV clinic are already pregnant.

Whilst pregnancy rates were higher amongst those who presented for having consented to sex as a minor, use of the emergency contraceptive pill and request for TOPs were lower than for those who were raped. This may be linked to the fact that a TOP is only legal under exceptional circumstances (this includes the case of rape, incest, serious threat to the mothers’ life or if the child is likely to be born with a handicap). Minors who reported to consent to sex are not allowed to access a TOP, under the Termination of Pregnancy Act (2003—Chapter 15:10), which specifies that a provincial magistrate may approve a TOP for pregnancies of less than 10 weeks with the possibility of exceptional authorisations up to 28 weeks at any stage before or after trial is commenced or finalized. For the majority of cases requesting TOP, there was no record in the case file regarding whether a TOP was carried out—either the person was LTFU or the act was not recorded. Some may not have been able to go through with the TOP either due to presenting late; having lost the court case; or a mismatch between the gestation and the alleged date of the incident. Some few cases may have gone through with a TOP, and some LTFU cases may have accessed an illegal abortion, while some may have opted to keep the baby, either on their own or with the perpetrator. The proportion of clients who requested and received a TOP at the clinic was very low. Similarly 20% of the clients eligible for EC did not take it. This might indicate that further education and sensitisation would be warranted on EC as a method of contraception.

Overall, only half the clients who came to the clinic had at least one follow-up visit. Rape victims were slightly more likely to seek follow up care than clients who consented to sex as a minor. Possible reasons could be that minors who are at odds with their parents over a relationship may be sent to their rural homes to live with relatives, or that adolescents might move away to live with the man they had sex with, away from the prying eyes of those around them, or that they don’t feel they need to come back. Of those eligible for PEP, 80% of minors who consented to sex vs 93% of clients who were raped took PEP. Here too, reasons may be varied, with some clients refusing to take PEP upon discovering that these are the same drugs as ART, leading to worries about taking the drugs home to be seen by their families; and others refusing PEP because they plan to go back to the same man. It is not known whether some clients who were lost to follow-up chose to be followed up, including for HIV testing, at their local clinic.

A number of adolescents who presented to the clinic for relationships involving consensual sex were likely to continue the relationship in some way after they or their parents made a complaint of sexual abuse with the police or at the clinic. Of the clients who presented to the clinic, some likely went on to have a child with the perpetrator if they were pregnant, and/or to marry the perpetrator. This was also the case for a number of minors whose initial statement was that they had been forced to have sex, but then later started a consensual relationship. It is probable that the clients presenting to the clinic represent the tip of the iceberg, as presentation was usually done following some sort of ‘emergency’–it is likely that there are a large number of adolescents engaging in sexual relationships of this kind.

Zimbabwe has experienced high rates of economic migration death related to HIV, and high numbers of HIV orphans meaning that traditional family structure has been altered [13]. This may leave some young girls to grow up without having adequate guidance from close family, and so may be relatively ill-informed when it comes to issues around their own bodies and their sexuality. These changes have coincided with increases in availability of new technology which provides young people with plenty of unfiltered information through internet and media regarding sex. Furthermore, a qualitative study in Harare and Chinoyi found that the use of Facebook by youths undermined both the parent-child relationship, and the parents’ ability to control or guide what their child did [14]. Teenagers in Zimbabwe, much like in other countries, are also subject to peer pressure to adhere to stereotyped sexual norms—i.e. for men to prove their manhood by having sex, and young women to be submissive and not to discuss sex, leaving them less able to refuse [15]. These roles can become exacerbated when teenagers are under the influence of drugs or alcohol. For example, a study carried out amongst university students found that the most commonly reported reason for unwanted sexual intercourse was impaired judgement due to alcohol [16].

To ensure safe access to sexual and reproductive health services, adolescents should have the right to consent to services that are confidential, private and where health workers are non-judgmental [9]. Continued efforts are needed to ensure adolescents have access to high quality and tailored services provided by nurses who have been trained to deal with this specific population, to enable them to protect themselves from sexual and reproductive health problems [1]. Unwanted pregnancies could be prevented by ensuring adolescents have access to information on sexuality and family planning, offering counselling in times of crises, supporting adolescents to build good social and decision making skills, all within a supportive environment [3].

The study had a number of limitations. Firstly, it was a facility-based review: clients who did not seek care, or who sought care at other clinics were not included. Such clients may have been very different from those presenting at the MSF clinic, thus introducing a possible bias. In particular, clients who experienced physical violence and presented first with the police would likely be referred to a doctor first, where a physical assault affidavit could be filled out. Male victims of rape, especially by another man may also be underrepresented due to the stigma of reporting this event, especially in a country where sodomy is illegal. Secondly, the analysis was retrospective: only pre-existing categories of variables could be used, which may not have been ideally suited for research purposes. For example, the variable “cause of delay in consultation” only had broad options such as ‘fear’ or ‘family negotiation’. Thirdly, some clients in this dataset may have experienced more than one type of event, but as only one incident could be registered, only the event that the client or guardian considered to be the primary event was registered. For example, for minors who said they were raped at first instance and then consented to a sexual relationship, the classification would depend on the description by the client. Finally, presentation within 72 hours means presentation within 72 hours of the last event, which in the case of multiple events inadequately describes the care seeking behaviour.

## Recommendations

### Medical

- To scale up access to provision of adolescent sexual and reproductive health education and information materials for adolescents before they consent to sex, to include:
  - To understand a healthy relationship, both the right to have consensual sex, and to refuse sex
  - To understand SRH problems and risks associated with early sexual debut and early pregnancy
  - To acquire decision-making skills and skills to negotiate safe and appropriate behaviour from their partner
- Prevention and care: Scale up access to HIV testing, care and treatment, PEP, STI screening and treatment, and other sexual and reproductive health care for adolescents
- Scale up access to all methods of family planning according to the FP guidelines of Zimbabwe: “all adolescents who are sexually active should be offered a contraceptive method of their choice”
- To increase access to adolescent sexual and reproductive health services through scaling up the number of adolescent-friendly services (confidential, private, non-judgemental) in health facilities with adolescent-friendly trained health personnel
- To encourage youths to visit a clinic as a couple to ascertain their HIV and STI status at an early stage and to identify whether they are sero-discordant

### Legal

- Improvements in the legal process regarding under age sex and rape would help improve safe access to SRH for adolescents
- To inform adolescents and adults that care can be sought for SGBV from medical providers, without seeking legal or police support first
- To discuss with legal stakeholders on what constitutes an “exceptional case” for accessing a TOP, and whether the law could be made safer to ensure fewer women opt for unsafe abortions

### Health promotion

- Increased use of new technology, using an interactive platform e.g. mobile phones (SMS, Whatsapp, Facebook…) and other social media would be helpful to improve knowledge regarding sex and rape.
- Radio, television, drama and door-to-door campaigns have been successfully carried out in the Mbare SGBV project in the past, and should continue to be scaled up.
- Group discussions with guardians, parents and community on ASRH services and support in case of rape (medical, social, legal)
- To disseminate information in a wide variety of forums that directly reaches out to children (in and out of school), parents, guardians and communities
- Integration of SGBV prevention messages into school-based programmes addressing SRH

## Conclusion

The experiences of those who consent to sex as a minor and those that have experienced forced sex are very different at the Mbare SGBV clinic. The standardised SGBV medical response does not fully meet the needs of minors who have consented to sex. Clients who present for having consented to sex as a minor might benefit more from being offered long-term family planning or being assessed as a sero-discordant couple rather than PEP and ECP, which may be more relevant for clients who have been raped. However, more provision of health care is needed for minors to ensure they have access to enough information and protection from HIV, other STIs and unwanted pregnancy, before they decide to engage in sexual intercourse, rather than as an emergency at an SGBV clinic.
